# Sample size calculation for randomized selection trials with a time‐to‐event endpoint and a margin of practical equivalence

**DOI:** 10.1002/sim.9490

**Published:** 2022-06-10

**Authors:** Hakim‐Moulay Dehbi, Andrew Embleton‐Thirsk, Zachary Ryan McCaw

**Affiliations:** ^1^ Comprehensive Clinical Trials Unit University College London London UK; ^2^ Insitro South San Francisco California USA

**Keywords:** early phase trials, randomization, sample size calculation, selection trials

## Abstract

Selection trials are used to compare potentially active experimental treatments without a control arm. While sample size calculation methods exist for binary endpoints, no such methods are available for time‐to‐event endpoints, even though these are ubiquitous in clinical trials. Recent selection trials have begun using progression‐free survival as their primary endpoint, but have dichotomized it at a specific time point for sample size calculation and analysis. This changes the clinical question and may reduce power to detect a difference between the arms. In this article, we develop the theory for sample size calculation in selection trials where the time‐to‐event endpoint is assumed to follow an exponential or Weilbull distribution. We provide a free web application for sample size calculation, as well as an R package, that researchers can use in the design of their studies.

## 1 INTRODUCTION

In selection trials two or more potentially active treatment options (eg, different dosage levels, schedules, or active compounds) are compared in a randomized fashion. These trials are applicable when a standard of care does not exist, for example in rare cancers.^1^ The selected option may become the standard of care or a backbone treatment with which various combination strategies are explored in future clinical trials.

Selection trials have traditionally used a binary endpoint as primary outcome variable, such as objective response rate (ORR) in oncology. However, time‐to‐event endpoints have recently been used in selection trials. For example, the FAME trial^2^ in lung adenocarcinoma is currently comparing fast‐mimicking diet and fast‐mimicking diet plus metformin (in addition to platinum‐pemetrexed chemotherapy in both arms), using progression‐free survival (PFS) as the primary endpoint. The NARLAL trial^3^ in locally advanced non‐small cell lung cancer (NSCLC) compared two doses of radiotherapy, 60 Gy and 66 Gy, concomitant with a fixed dose of oral vinorelbine, using local progression‐free survival at 9 months as primary endpoint. The 66 Gy dose was selected given that it was associated with a 59% local progression‐free survival at 9 months compared to 54% in the 60 Gy arm.

Selection trials often use a “pick‐the‐winner” decision rule. The treatment option that is chosen is simply the one with the highest efficacy numerically.^4^ When selecting among competing options, efficacy may not be the only relevant factor, especially when the observed efficacy levels are very similar. To remedy this, the concept of a margin of practical equivalence (MPE) in selection trials was first described by Sargent and Goldberg,^5^ and then more fully characterized by Dehbi and Hackshaw.^6^ The margin determines the amount of additional efficacy that is required so that one of the options can be selected on efficacy grounds alone (assuming that the toxicity profile remains acceptable, and that the observed efficacy is satisfactory with respect to some external/historical minimum threshold). If the observed efficacy levels are within the pre‐specified MPE, then additional dimensions such as toxicity, quality of life, and cost are evaluated to make the selection. This approach was followed by InterAAct,^1^ which was a study in squamous cell carcinoma of the anus that compared the doublet combination of cisplatin and 5‐fluorouracil (FU) with carboplatin and paclitaxel. The decision rule was based on efficacy, and if efficacy was non‐differential, toxicity and then quality of life would be examined. The results did not show a marked difference in efficacy: ORR was 57% (95% confidence interval (CI), 39.4% to 73.7%) for cisplatin plus FU vs 59% (95% CI, 42.1% to 74.4%) for carboplatin plus paclitaxel. However there were fewer adverse events with carboplatin plus paclitaxel and an extended PFS and overall survival (OS) compared to cisplatin plus FU. For this reason InterAAct recommended carboplatin plus paclitaxel as the chemotherapy backbone for this tumor type.

**FIGURE 1:**
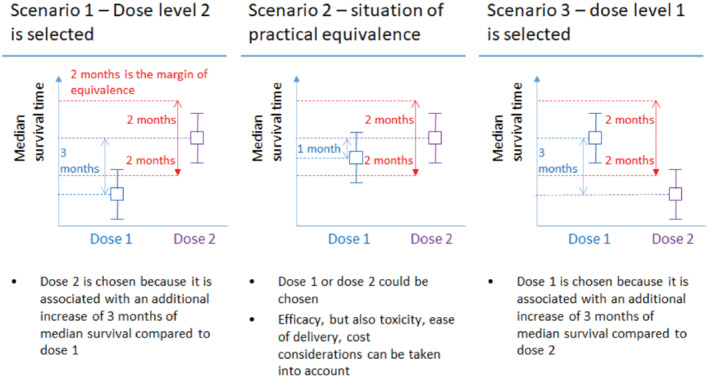
Conceptual overview of the three possible scenarios in a selection trial with a MPE where the primary endpoint is overall survival. The squares correspond to the observed median survival times, and the whiskers correspond to the confidence intervals

Figure 1 presents the three possible scenarios at the end of a selection trial with a MPE. At design‐stage, the sample size for selection trials can be determined by assuming that one of the treatment options (eg, one of the two dosage levels, usually the higher dosage level) is superior to the other(s). A threshold is then defined, for example 80% or 90%, and the sample size is numerically evaluated so that the chance of selecting the superior treatment is greater or equal to the threshold. A sample size calculator for binary endpoints (eg, ORR or PFS dichotomized at a specific time point) was developed by Dehbi and Hackshaw,^6^ and is available online at https://hakdehbi.shinyapps.io/randomised_phase_2_margin_equiv/. One of the advantages of ORR over a time‐to‐event endpoint, such as OS, is that ORR can be measured after a few cycles of treatment. In contrast, depending on the tumor prognosis OS data may take years to mature. This being said, in advanced and/or rare cancers where the prognosis is poor, median OS may be expressed in weeks or months. In this article, we extend the methodology of our previous publication^6^ to time‐to‐event endpoints. The aim is to enable selection trials having OS or PFS as their primary endpoint. More generally, survival is arguably among the most important outcomes for cancer patients and their families. Given that ORR has not been established as a valid surrogate for OS,^7^, ^8^, ^9^, ^10^, ^11^, ^12^ there is a general tendency for early phase or selection trials in oncology to use time‐to‐event endpoints whenever logistically feasible, as seen for example with FAME^2^ and NARLAL.^3^

The organization of the article is as follows. In Section 2, we develop the statistical theory of sample size calculation for time‐to‐event endpoints in selection trials. In Section 3, we revisit the sample size calculation of the InterAAct trial, to show the difference in sample size if a time‐to‐event endpoint (OS) had been employed instead of ORR. We use this case study to illustrate our web application, which can be used to calculate sample sizes for a variety of input parameters. In Section 4, we introduce the R package PracticalEquiDesign that we developed specifically for the design of selection trials with time‐to‐event endpoints. We conclude the article with final remarks in Section 5.

## 2 METHODOLOGICAL DEVELOPMENTS FOR SAMPLE SIZE CALCULATION

As depicted in Figure 1, for a comparison of two dosage levels, the trial may end in one of three possible states. From the perspective of dose 2, these states may be described as superiority, practical equivalence, and inferiority (to dose 1), respectively. Let $p_{\sup}$, $p_{\text{equi}}$, $p_{\inf}$ denote the probabilities of these three states across hypothetical repetitions of the trial, with $p_{\sup}+p_{\text{equi}}+p_{\inf}=1$. Now suppose dose 2 is in fact more efficacious, and define π as the probability that the more efficacious dose is ultimately selected. This may occur in one of two ways:

1. At the end of the trial, the observed median for dose 2 may exceed that of dose 1 by more than the MPE, which occurs with probability $p_{\sup}$.
2. Alternatively, the observed medians may fall within the MPE, which occurs with probability $p_{\text{equi}}$, and dose 2 may be selected on the basis of other considerations, such as toxicity and quality of life.

Lacking detailed information on the probability that dose 2 will be selected if the state of practical equivalence is reached, we apply the principle of indifference by supposing, for the sake of design, that dose 2 will be selected with probability 0.5 under practical equivalence. We may therefore write $\pi =p_{\sup}+0.5p_{\text{equi}}$. In the following, given a MPE and hypothesized medians for the two doses, we show how to determine the sample size needed to ensure π exceeds some threshold (eg, 80%) first under an exponential time‐to‐event model, then under a more‐general Weibull model.

### 2.1 Sample size for randomized selection trials with exponentially distributed time‐to‐event endpoint

We begin with the simplest case of no censoring, where for each participant the time‐to reach the event of interest is known. Consider a selection trial comparing two dosage levels, denoted as levels 1 and 2 for simplicity, and that the survival times T are exponentially distributed, that is, $T_{1}∼\mathrm{Exp}\;(\lambda_{1})$ and $T_{2}∼\mathrm{Exp}\;(\lambda_{2})$, $\lambda_{i}>0$, i=1,2, with exponential density $f(t)=\lambda e^{-\lambda t},t>0$.

Let us assume that $\lambda_{1}>\lambda_{2}$, which implies that the mean and median survival time with dose 2 is greater than with dose 1, that is, ${\bar{T}}_{1}=1/\lambda_{1}<\bar{T_{2}}=1/\lambda_{2}$, and $\tilde{T_{1}}=\ln(2)/\lambda_{1}<\tilde{T_{2}}=\ln(2)/\lambda_{2}$.

When there is no censoring the estimator $\hat{\bar{T}}$ of the mean survival time is unbiased. As the mean of a random sample of size n of exponentially distributed observations with rate parameter λ, $\hat{\bar{T}}$ follows a Gamma distribution whose parameters depend on the sample size and the underlying rate: $\hat{\bar{T}}∼\text{Gamma}\;(\text{shape}=n,\text{scale}=\frac{1}{n\lambda })$.

Considering the special case without MPE first, the question is then, for a given threshold Q what sample size is needed to ensure that:

$$\mathrm{Pr}\;(\hat{{\bar{T}}_{1}}<\hat{{\bar{T}}_{2}})\geq Q.$$

In this context, $\mathrm{Pr}\;(\hat{{\bar{T}}_{1}}<\hat{{\bar{T}}_{2}})$ is $p_{\sup}$.

Equivalently, one can consider:

$$\mathrm{Pr}\;(\hat{{\bar{T}}_{1}}-\hat{{\bar{T}}_{2}}<0)\geq Q,$$

to make use of the Gamma difference distribution (GDD).^13^ In a generic way the GDD is specified as follows. Let $X_{1}$ and $X_{2}$ be two independent random Gamma variables such that $X_{1}∼\text{Gamma}\;(\alpha_{1},\beta_{1})$ and $X_{2}∼\text{Gamma}\;(\alpha_{2},\beta_{2})$, where $\alpha_{i}>0$, $\beta_{i}>0$, i=1,2. The random variable $X_{1}-X_{2}∼\text{GDD}\;(\alpha_{1},\alpha_{2},\beta_{1},\beta_{2})$. With Γ(·) denoting the standard gamma function and the lower incomplete gamma function defined as:

$$\gamma (\alpha ,y)=\int_{0}^{y}t^{\alpha -1}e^{-t}\;dt,$$

the cumulative distribution function F of the $\text{GDD}\;(\alpha_{1},\alpha_{2},\beta_{1},\beta_{2})$ is:

$$F(t)=\frac{\beta_{2}^{\alpha_{2}}}{\Gamma (\alpha_{1})\Gamma (\alpha_{2})}\int_{\max[0,-t]}^{\infty }x^{\alpha_{2}-1}e^{-\beta_{2}x}\gamma (\alpha_{1},\beta_{1}(x+t))\;dx\;\;\;(t\in \mathbb{R}).$$

**FIGURE 2:**
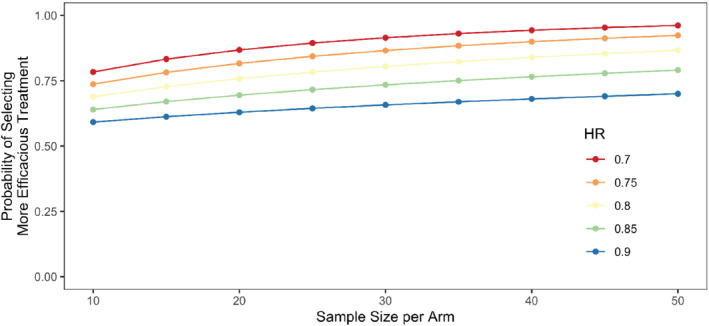
Probability of selecting the more efficacious treatment as a function of the sample size and the hazard ratio (HR), comparing dose 2 vs dose 1, when the data are exponentially distributed without censoring. For all five curves $\lambda_{1}$ = 0.1

Under a 1:1 randomization of n patients to each of the two dosage levels in our selection trial, the parameters of the GDD are $\alpha_{1}=\alpha_{2}=n$, $\beta_{1}=(1/\lambda_{1})/n$ and $\beta_{2}=(1/\lambda_{2})/n$. Figure 2 displays the probability of observing a difference in the mean survival time as a function of the sample size per arm and the hazard ratio ($\lambda_{2}/\lambda_{1}$) under an exponential time‐to‐event distribution without censoring.

If a MPE is used, the more efficacious treatment option is chosen in two distinct situations:

1. The observed difference between the observed mean survival time in the more efficacious dose level compared to the other level is greater than the margin;
2. The most efficacious treatment is chosen in a situation of practical equivalence where we only have two options, which has a probability of 50% assuming that the other considerations of interest (eg, toxicity, cost, QoL) are unrelated to efficacy.

Define $\pi_{n}(\text{MPE})$ as the probability of selecting the more efficacious treatment under sample size n and MPE (eg, 3 months of survival). For the exponential distribution:

(1) $$\pi_{n}(\text{MPE})=\mathrm{Pr}\;(\hat{{\bar{T}}_{1}}-\hat{{\bar{T}}_{2}}<-\text{MPE})+0.5\times \mathrm{Pr}\;(-\text{MPE}\leq \hat{{\bar{T}}_{1}}-\hat{{\bar{T}}_{2}}<\text{MPE}).$$

Here, $\mathrm{Pr}\;(\hat{{\bar{T}}_{1}}-\hat{{\bar{T}}_{2}}<-\text{MPE})$ is $p_{\sup}$, the probability that the trial ends with dose 2 superior, and $\mathrm{Pr}\;(-\text{MPE}\leq \hat{{\bar{T}}_{1}}-\hat{{\bar{T}}_{2}}<\text{MPE})$ is $p_{\text{equi}}$, the probability that the trial ends in a state of practical equivalence. The sample size n necessary to ensure $\pi_{n}(\text{MPE})$ exceeds some threshold Q is easily calculated by means of the GDD.

We now turn to the case of survival data with right censoring. Because of the presence of censoring, instead of observing all event times $T_{1},\ldots ,T_{n}$, we observe $(U_{i},\delta_{i}),i=1,\ldots ,n$, where $U_{i}=\min(T_{i},C_{i})$, $\delta_{i}=𝕀(T_{i}\leq C_{i})$ and $C_{i}$ is the random potential censoring time. We assume non‐informative right censoring, that is, $T_{i}⊥\;⊥C_{i}$. Given that the $(U_{i},\delta_{i}),i=1,\ldots ,n$, are i.i.d and exponentially distributed, the likelihood is:

$$L(\lambda )=\overset{n}{\underset{i=1}{\prod }}{\left(\lambda e^{-\lambda u_{i}}\right)}^{\delta_{i}}{\left(e^{-\lambda u_{i}}\right)}^{1-\delta_{i}}=\lambda^{r}e^{-\lambda W},$$

where $r=\sum_{n=1}^{n}\delta_{i}$ and $W=\sum_{n=1}^{n}u_{i}$. The first and second derivatives of the log‐likelihood are $\frac{\partial \ln L(\lambda )}{\partial \lambda }=\frac{r}{\lambda }-W$ and $\frac{\partial^{2}\ln L(\lambda )}{\partial^{2}\lambda }=-\frac{r}{\lambda^{2}}$ respectively. The observed information Î(λ), defined as the negative of the second derivative of the log‐likelihood, is $\frac{r}{\lambda^{2}}$. Given that r is the number of uncensored observations, which follows a binomial distribution with probability of non‐censoring p, it follows that for a sample size of n, $Î(\lambda )=\frac{np}{\lambda^{2}}$. Using the central limit theorem (CLT), the sampling distribution of $\hat{\lambda }=\frac{r}{W}$ converges, as n→∞, to $\hat{\lambda }∼N\{\lambda ,I^{-1}(\lambda )\}=N(\lambda ,\frac{\lambda^{2}}{np})$. Applying the delta method, the log of $\hat{\lambda }$ is also normally distributed, $\ln\hat{\lambda }∼N(\ln\lambda ,\frac{1}{np})$. Since the sampling distribution of $\ln\hat{\lambda }$ is normally distributed, the distribution of $\ln\hat{\lambda_{2}}-\ln\hat{\lambda_{1}}$ is also normally distributed, $\ln\hat{\lambda_{2}}-\ln\hat{\lambda_{1}}∼N(\ln\lambda_{2}-\ln\lambda_{1},\frac{2}{np})$. The ratio $\frac{\lambda_{2}}{\lambda_{1}}$ is the familiar hazard ratio (HR), which means that the previous expression reduces to $\ln\hat{\lambda_{2}}-\ln\hat{\lambda_{1}}∼N\{\ln(HR),\frac{2}{np}\}$. These results allows us to calculate the required sample size by making use of the standard normal distribution.

Assuming $\lambda_{1}>\lambda_{2}$, we may re‐express (1) as:

$$\pi_{n}({\text{MPE}}_{\lambda })=\mathrm{Pr}\;(\hat{\lambda_{2}}-\hat{\lambda_{1}}<-{\text{MPE}}_{\lambda })+0.5\times \mathrm{Pr}\;(-{\text{MPE}}_{\lambda }\leq \hat{\lambda_{2}}-\hat{\lambda_{1}}<{\text{MPE}}_{\lambda }),$$

where ${\text{MPE}}_{\lambda }=\rho (\lambda_{1}-\lambda_{2})$ and ρ∈[0,1], then calculate the sample size required such that $\pi_{n}({\text{MPE}}_{\lambda })$:

$$\hat{n}=\hat{n}({\text{MPE}}_{\lambda },Q)=\arg\min\{n\in \mathbb{N}:\pi_{n}({\text{MPE}}_{\lambda })\geq Q\}.$$

**FIGURE 3:**
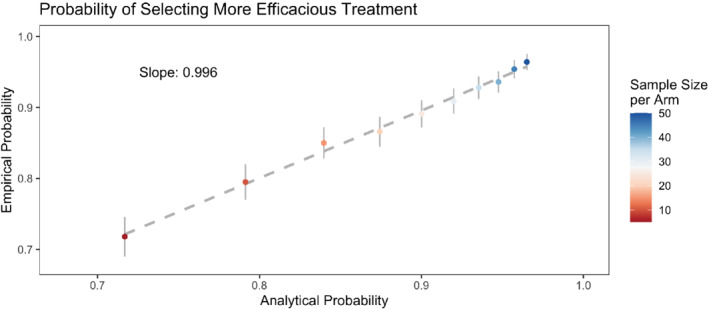
Calibration plot for the probability of selecting the more efficacious treatment comparing analytical and empirical probabilities, across $10^{3}$ simulation replicates, for sample sizes between 5 and 50 patients per dosage level, for $\tilde{T_{1}}$ = 6 months and $\tilde{T_{1}}$ = 9 months, using 20% censoring and a MPE of length 0

The above results are asymptotic. We verified their calibration empirically, across 1000 simulation replicates, for small sample sizes ranging from 5 to 50 patients per dosage level. This is shown on Figure 3, assuming 20% censoring, median survival times of 6 and 9 months, and no MPE (ie, a MPE of length 0). The empirical probability is the frequency with which the estimated $\lambda_{1}<\lambda_{2}$ across simulations while the analytical probability is that obtained via asymptotic calculations.

### 2.2 Sample size for randomized selection trial with Weilbull distributed time‐to‐event endpoint

Now suppose the underlying time‐to‐event for the kth treatment arm, k=1,2, follows a Weibull distribution with shape parameter $\alpha_{k}$ and rate parameter $\lambda_{k}$. For t>0, we suppose the density is parameterized as $f(t)=\alpha_{k}\lambda_{k}{\left(\lambda_{k}t\right)}^{\alpha_{k}-1}e^{-{\left(\lambda_{k}t\right)}^{\alpha_{k}}}$. Also let $\mu_{k}$ denote the median time‐to‐event in arm k. In the case of the exponential distribution, which is characterized by a single rate parameter, ordering two distributions with respect to their rate parameters was equivalent to ordering them with respect to their median event times. In particular, since the median time‐to‐event of the exponential distribution is $\mu =\lambda^{-1}\ln2$, if the event rate is greater in arm 1 ($\lambda_{1}>\lambda_{2}$), then the median time‐to‐event is greater in arm 2 ($\mu_{1}<\mu_{2}$). Unlike the exponential distribution, the Weibull is characterized by two parameters: a shape and a rate. However, generalizing from the exponential, Weibull distributions may still be ordered by comparing their median event times. For a Weibull distribution with shape α and rate λ, the median time to event is $\mu =\lambda^{-1}{\left(\ln2\right)}^{1/\alpha }$. We describe dosages 1 and 2 as practically equivalent if the difference in the medians is within the MPE, $|\mu_{2}-\mu_{1}|<\text{MPE}$, and dosage 2 as superior if the median of dosage 2 exceeds that of dosage 1 by more than the margin of practical equivalence, $\mu_{2}-\mu_{1}>\text{MPE}$.

Since in practice $\mu_{1}$ and $\mu_{2}$ are unknown, we next consider estimation of the median event time in each treatment arm under non‐informative random right censoring. For a given arm, the data consists of n tuples of the form $\left(U_{i},\delta_{i}\right)$. The right censored Weibull likelihood is:

(2) $$L(\alpha ,\lambda )=\overset{n}{\underset{i=1}{\prod }}\{\alpha \lambda^{\alpha }u_{i}^{\alpha -1}{\}}^{\delta_{i}}e^{-{\left(\lambda u_{i}\right)}^{\alpha }}.$$

Given α, the maximum likelihood estimator (MLE) of λ is:

$$\hat{\lambda }(\alpha )={\left(\frac{\sum_{i=1}^{n}u_{i}^{\alpha }}{\sum_{i=1}^{n}\delta_{i}}\right)}^{-1/\alpha }.$$

Substituting $\hat{\lambda }(\alpha )$ for λ in (2) generates a profile likelihood $L_{p}(\alpha )=L\{\alpha ,\hat{\lambda }(\alpha )\}$, which may be optimized numerically to obtain the MLE $\hat{\alpha }$ of α. By the invariance principle, the MLE of the median is then $\hat{\mu }={\hat{\lambda }}^{-1}{\left(\ln2\right)}^{1/\hat{\alpha }}$, where $\hat{\lambda }=\hat{\lambda }(\hat{\alpha })$. A large sample SE for $\hat{\mu }$ may be obtained using the delta method, the details of which are provided in the Supplementary Material.

For sample size estimation, we employ the large sample approximation ${\hat{\mu }}_{k}\overset{\cdot }{˜}N(\mu_{k},{\hat{\sigma }}_{\mu ,k}^{2})$, where ${\hat{\mu }}_{k}$ is the estimated median time‐to‐event in treatment arm k under the Weibull model, and ${\hat{\sigma }}_{\mu ,k}$ is the corresponding SE. Note that ${\hat{\sigma }}_{\mu ,k}$ is implicitly ${𝒪}_{p}(n^{-1/2})$. For a given margin of practical equivalence MPE, the probability of choosing the more efficacious dosage level is:

(3) $$\begin{array}{ll} \pi_{n}(\text{MPE}) & \equiv \mathrm{Pr}({\hat{\mu }}_{2}-{\hat{\mu }}_{1}>\text{MPE})+\frac{1}{2}\times \mathrm{Pr}(|{\hat{\mu }}_{2}-{\hat{\mu }}_{1}|\leq \text{MPE})\\ & =1-\frac{1}{2}\Phi \left\{\frac{\text{MPE}-(\mu_{2}-\mu_{1})}{\sqrt{{\hat{\sigma }}_{\mu ,1}+{\hat{\sigma }}_{\mu ,2}}}\right\}-\frac{1}{2}\Phi \left\{\frac{-\text{MPE}-(\mu_{2}-\mu_{1})}{\sqrt{{\hat{\sigma }}_{\mu ,1}+{\hat{\sigma }}_{\mu ,2}}}\right\}, \end{array}$$

where Φ is the standard normal distribution function. Using $\pi_{n}(\text{MPE})$, the sample size necessary to ensure the more efficacious dosage is selected with probability at least Q is:

$$\hat{n}=\hat{n}(\text{MPE},Q)=\arg\min\{n\in \mathbb{N}:\pi_{n}(\text{MPE})\geq Q\}.$$

To verify the power calculation in (3), for each sample size n∈{5,10,…,100}, $R=10^{3}$ Weibull data sets were simulated with shape and rate parameters $\left(\alpha_{1},\lambda_{1}\right)$ and $\left(\alpha_{2},\lambda_{2}\right)$ for dosage levels 1 and 2, respectively. For each treatment arm k∈{1,2}, the MLE of the median ${\hat{\mu }}_{k}$ and the corresponding SE ${\hat{\sigma }}_{\mu ,k}$ were obtained using the Temporal ^14^ package in R. For a specified MPE, the probability of choosing the more efficacious treatment $\pi_{n}(\text{MPE})$ was calculated in two ways:

1. *Analytically*, using the large sample approximation in Equation (3).
2. *Empirically*, by averaging $𝕀({\hat{\mu }}_{2}-{\hat{\mu }}_{1}>\text{MPE})+0.5\times 𝕀(|{\hat{\mu }}_{2}-{\hat{\mu }}_{1}|\leq \text{MPE})$, where 𝕀(·) denote the indicator function, across simulation replicates.

**FIGURE 4:**
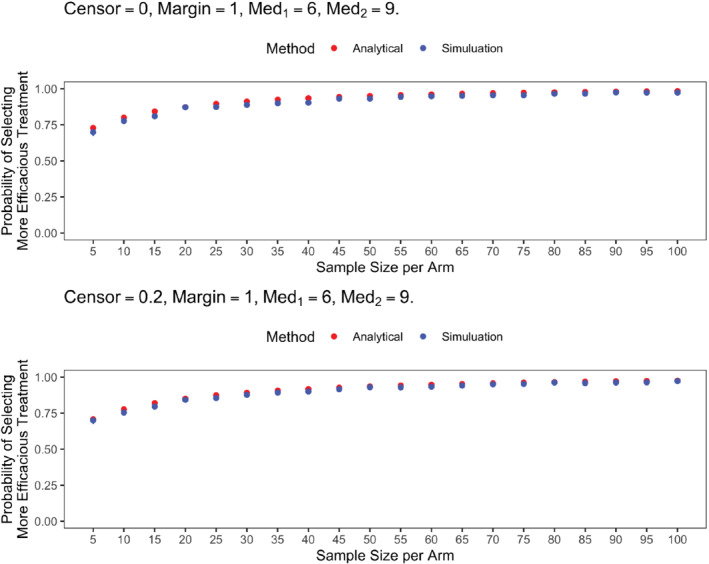
Analytical vs empirical probabilities of selecting the more efficacious treatment under Weibull event times

Figure 4 presents a comparison of the selection probability curves, calculated analytically (red) and empirically (blue), in the case of two exponential distributions (ie, $\alpha_{1}=\alpha_{2}=1$) with median event times of $\mu_{1}=6$ (eg, months) and $\mu_{2}=9$. The MPE was 1 month. The close correspondence of the two curves demonstrates that the analytical (asymptotic) calculation is accurate, even at small sample size. As expected, the agreement between the analytical and empirical calculations further improves as sample size increases.

Additional simulation settings are presented in the Supplementary Material. Of note is the setting of no difference in the median event times of the two arms (Figures S1 and S2), in which case the probability of selecting either treatment is 0.5 and the analytical calculation is exact. Also of interest is the setting of a 1 month difference in the median event times between the two arms (Figures S3 and S4). For this setting, when the MPE is set to 0, the probability of selecting the more efficacious treatment tends towards 1.0 with increasing sample size. When the MPE is set to 2, in which case the anticipated difference in medians is within the margin, the probability of selecting the more efficacious treatment tends towards 0.5 with increasing sample size. In general, when the anticipated difference in medians falls within the MPE, the probability of selecting the more efficacious treatment is expected to decline towards 0.5 with increasing sample size. This occurs because, at small sample sizes, the more efficacious treatment can by chance outperform the comparator by more than the MPE, but as sample size increases, the difference in medians will with increasing probability fall within the MPE. It should be noted that designing a trial in which the anticipated difference in medians falls within the MPE is of little practical interest. For the setting where the anticipated difference in medians exceeds the MPE, the probability of selecting the more efficacious treatment will increase monotonically with sample size.

## 3 INTERAACT REDESIGNED WITH OVERALL SURVIVAL AS PRIMARY ENDPOINT

In InterAAct^1^ the primary endpoint was the ORR, which was assumed to be 40% in the cisplatin plus FU arm. The clinically relevant difference in ORR between arms was defined as 10 percentage points. Based on this, 40 patients per arm were required for an 80% probability to detect a 10% difference in ORR between the arms.

**FIGURE 5:**
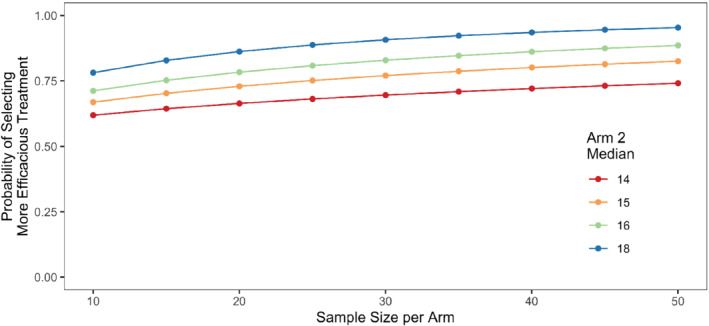
Probability of selecting the more efficacious treatment in InterAAct with overall survival as primary endpoint, using a median survival of 12 months in the reference arm and a 1 month margin of practical equivalence

At the time InterAAct was designed,^1^, ^15^ a retrospective case series analysis showed a median survival of 12 months for carboplatin plus paclitaxel in patients with advanced anal cancer.^16^ Assuming a difference comprised between 2 and 6 months between the superior arm and the other arm, with a reference at 12 months for the median OS of the inferior arm, Figure 5 below presents the probability of correct selection of the superior arm depending on the sample size, assuming an exponential survival model.

It appears that a sample size of 40 patients per arm provides 80% power if there is a difference of 3 months in median OS. This calculation includes a MPE of 1 month, and assumes a censoring rate of 20%. If the difference was assumed to be larger between the arms, for example 4 months, then 25 patients per arm would provide 80% chance to correctly rank the two treatments.

The main learning from this analysis is that for the same sample size of 40 patients InterAAct could have used a more definitive time‐to‐event endpoint such as OS.

The trial reported a pronounced difference between the two treatments: median OS was 12.3 months for cisplatin plus FU compared with 20 months for carboplatin plus paclitaxel. If this difference of approximately 8 months in median survival times had been used at the design stage, with a MPE of 1 month, 20 patients per arm would have provided 90% power for identifying the superior arm.

**FIGURE 6:**
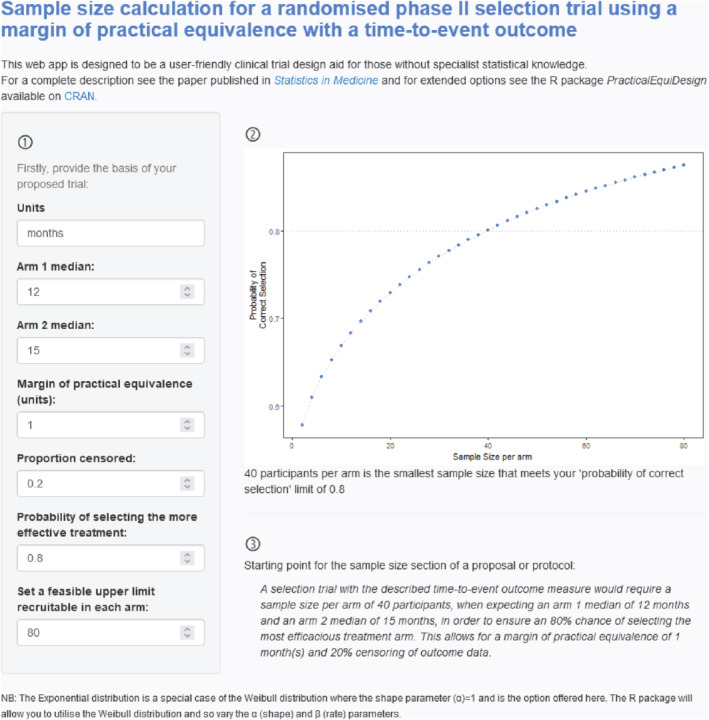
Illustrative sample size calculation using the web application in the case of the InterAAct trial

We developed a free web application that can be accessed at https://andyembleton.shinyapps.io/sample_size_tte_selection/. This has been designed to be a user‐friendly trial design aid for those without specialist statistical knowledge or those wishing to demonstrate design choices easily. It can be used to calculate the minimum sample size for a two‐arm selection trial for a variety of input parameters. These parameters can be easily varied to explore and demonstrate to collaborators their impact. Designing the InterAAct trial, as discussed in this section, the application could be utilized as shown in Figure 6. In the first, left‐hand side panel, we can enter the units to be used, the expected medians for the two arms, any MPE allowed in the design, the anticipated proportion of outcome data censored. In addition, the minimum acceptable probability of selecting the more effective treatment and a broad feasible upper limit to the arm can be entered. The right‐hand side illustrates the probability of correct selection over a range of sample sizes, with a reference line and a passage below the plot stating the minimum sample size required to meet the required threshold. Finally, a sample summary paragraph incorporating the chosen design is provided as a starting point for the sample size section of a research proposal or trial protocol.

## 4 ILLUSTRATION OF OUR R PACKAGE USING A HYPOTHETICAL TRIAL WITH WEIBULL‐DISTRIBUTED DATA

**FIGURE 7:**
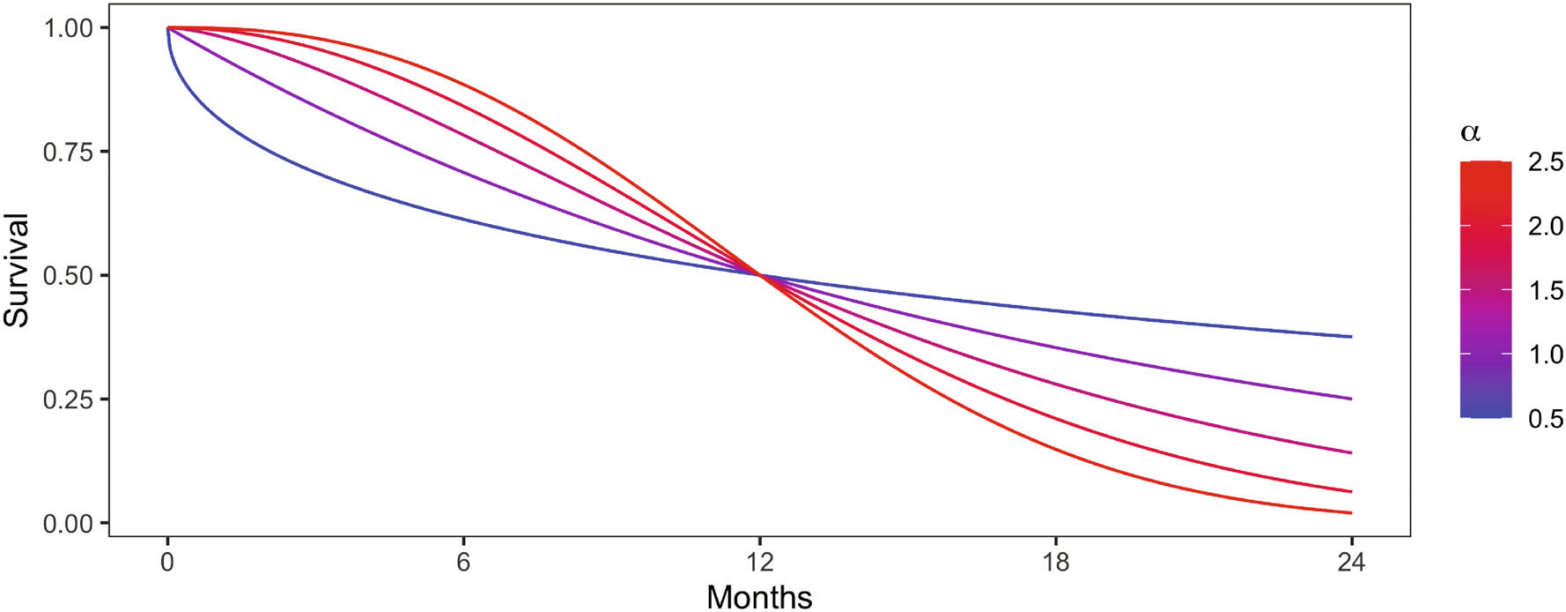
Possible survival curves for a Weibull distribution with a median of 12 months

Now consider designing a trial in which the median survival time in the reference arm is 12 months and follows a Weibull distribution has two, controlled by the shape parameter α and the rate parameter λ. Figure 7 depicts different Weibull distributions with a median of 12 months. α=1.0 (purple) corresponds to the exponential distribution. As α increases, the survival curve declines more quickly through the median. To specify a Weibull distribution uniquely, it suffices to set the survival probability at two distinct time points. The function WeibullSpec in the accompanying R package performs this calculation. For example, if the anticipated survival rate at 6 months is 80% and that at 12 months is 50%, the corresponding Weibull distribution has shape α=1.635 and rate λ=0.067.

Suppose $\alpha_{0}=1.635$ and $\lambda_{0}=0.067$ summarize the survival distribution in the reference arm, and that a new treatment is anticipated to extend median survival by 20% to 16 months. If the new treatment improves survival uniformly, such that the 80th percentile likewise improves by 20% (to 7.2 months), the Weibull distribution for the treatment arm will have $\alpha_{1}=1.419$ and $\lambda_{1}=0.048$. Assuming 20% censoring and a 2‐month MPE, a sample size of 15 patients per arm provides an 80% chance of selecting the more efficacious treatment. This calculation is performed by the SampleSize function in the accompanying R package. Suppose instead that the new treatment is effective only within a subset of the population, such that the median is extended to 16 months but the 80th percentile remains unchanged at 6.0 months. The corresponding Weibull distribution has $\alpha_{1}=1.156$ and $\lambda_{1}=0.046$. The number of patients needed per arm to select the better treatment with 80% probability increases to 21. The greater sample size requirement in this case compared to the previous is intuitive: although the median increased by the same amount (20%), the treatment that also improved the 80th percentile is clearly more beneficial, making its superiority easier to detect. This example demonstrates the utility of the Weibull distribution's added degree of freedom for exploring scenarios where a new treatment might differentially affect two points along the survival curve (eg, the median and 80th percentile).

As a final example, suppose again that the new treatment increases median survival from 12 to 16 months while leaving the 80th percentile unchanged at 6 months. If the MPE were decreased from 2 to 1 month, the sample size required to select the more effective treatment with 80% power would decrease from 21 to 18 patients per arm. Alternatively, if the MPE were increased from 2 to 3 months, the sample size requirement would increase from 21 to 33 patients per arm. The increase in required sample size with the MPE is a general trend. This occurs because, for the purpose of sample size calculation, the more effective treatment is only chosen with 50% probability if the difference in estimated medians falls within the margin. Thus, for a fixed sample size, as the width of the margin increases towards the true distance between the medians, the probability of selecting the more effective treatment steadily declines towards the hypothesized value of 50%. In practice, if the difference in medians falls within the margin, then treatment selection is not random but rather based on other considerations, such as toxicity. Nevertheless, for the purpose of sample size estimation, it is necessary to specify the probability of selecting the more efficacious treatment when a state of practical equivalence is reached.

## 5 FINAL REMARKS

In selection trials the objective is to recommend the most appropriate treatment option for further study. Unless there is a significant difference efficacy‐wise between options, the choice must take into account multiple dimensions in addition to efficacy. In order to achieve this in a formal way, the MPE can be set at the study design stage. A conversation among the research team would then take place about what dimensions need to be taken into account, in which order, and potentially with which weights. For example, in NEOSCOPE,^17^ a randomized trial of induction chemotherapy followed by either oxaliplatin/capecitabine (OxCap)‐ or carboplatin‐paclitaxel (CarPac)‐based chemoradiation as a pre‐operative regimen for resectable esophageal cancer, the protocol specified an algorithm with five steps, based on pathological complete response (pCR; the primary endpoint), operative mortality, and toxicities to make the decision.

Selection trials make most sense when there is no established standard of care. This was the case in InterAAct for squamous cell carcinoma of the anus,^1^ where the objective was to set a standard of care and establish the cytotoxic backbone treatment for future clinical trials. However, when there is a standard of care, seamless phase 2/3 trials, such as multi‐arm multi‐stage trials in which the phase 2 component consists of selecting experimental agents against the standard of care, make more efficient use of resources than conducting two distinct trials, the first being a selection trial without comparison to a standard of care and the second being the comparison of the selected agent with the standard of care. Another use of the discussed selection design is in early phase dose‐finding trials where two or more dosage levels may be compared in a randomized manner in the expansion part to establish the recommended phase 2 dose.^18^

The concept of type I error does not apply to sample size calculation in selection trials, as there is no internal comparator. This being said, studies can set a minimum efficacy threshold that a treatment option must pass in order to be taken forward. Such threshold can be established with respect to external/historical references. In NEOSCOPE,^17^, ^19^ the minimum response rate was defined as 15%. Only one of the two treatment arms, carboplatin‐paclitaxel (CarPac), achieved a pCR rate greater than 15%, with 29.3% among resected patients, while the pCR rate with OxCap was 13.9%. This observed difference of approximately 15 percentage points was large enough, combined with the fact that one of the two arms did not pass the minimum 15% bar pCR‐wise, that the decision to take forward CarPac did not involve operative mortality and toxicities.

In this article, we leverage parametric survival distributions to calculate the required sample size for a selection trial based on a time‐to‐event endpoint. In the absence of prior information about the shape of the survival curve, or if it is known from external evidence that the curves are reasonably well approximated by an exponential or Weibull model, then our approach is an appropriate starting point. The underlying hazard function is constant in an exponential model, and a monotonic function of time in a Weibull model. If however there is evidence that the hazard function might have a different shape, simulations can be used to calculate the required sample size, as it is possible to simulate complex survival curves for a variety of underlying hazard curves.^20^, ^21^ Time‐to‐event endpoints are frequently used in clinical trials, both in oncology and in other disease areas. In oncology, the relationship between ORR, PFS, and OS has been studied in multiple cancer types but is not established in general.^7^, ^8^, ^9^, ^10^, ^11^, ^12^ As an alternative to ORR, researchers have dichotomized time‐to‐event endpoints as, for example, in NARLAL.^3^ We have shown that with similar sample sizes one can study and compare the full survival distributions between the groups. Consequently, whenever logistically possible, it is important to design studies with a primary endpoint that is as close as possible to the interests of patients and which maximize the use of information. According to ICH‐E9, the International Council for Harmonization (ICH) guideline E9 Statistical Principles for Clinical Trials,^22^ ``The primary variable should be the variable capable of providing the most clinically relevant and convincing evidence directly related to the primary objective of the trial''.

We have developed a Web application (available freely at https://andyembleton.shinyapps.io/sample_size_tte_selection/) as well as an R package (called PracticalEquiDesign, available on CRAN) so that researchers can calculate the sample size that is required for a selection trial with a time‐to‐event endpoint for a variety of input parameters.

## CONFLICT OF INTEREST

The authors declare no potential conflict of interests.

## Supporting information

Data S1: Supplementary material

## Data Availability

Data sharing is not applicable to this article as no new data were obtained or analyzed in this study. We only used simulated data.
