# Supplementary material for: Sample size calculation for randomized selection trials with a time‐to‐event endpoint and a margin of practical equivalence
Source: Stat Med. 2022 Jun 10;41(20):4022–33. doi: 10.1002/sim.9490 (PMC9544500; doi:10.1002/sim.9490)
Supplement: Supplementary file 1 — Data S1: Supplementary material [file SIM-41-4022-s001.pdf]

Sample size calculation for randomised selection trials with a time-to-event endpoint and a margin of practical equivalence: Supplementary Material

May 26, 2022

1 Standard Error of the Weibull Median

Here we show how to derive an estimate for the standard error of the maximum likelihood estimator of Weibull median. As the procedure is the same for each, consider a single treatment arm. In the presence of non-informative random right censoring, the observed data are $U_i = \min(T_i, C_i)$, $T_i \sim \text{Weibull}(\alpha, \lambda)$, and $\delta_i = \mathbb{I}(T_i \leq C_i)$ for $i \in \{1, \cdots, n\}$. The right censored log likelihood is:

$$\ell(\alpha, \lambda) = \sum_{i=1}^{n} \delta_i \big\{ \ln \alpha + \alpha \ln \lambda + (\alpha - 1) \ln u_i \big\} - \lambda^{\alpha} u_i^{\alpha}.$$

The score equation for α is:

$$\mathcal{U}_\alpha = \frac{\partial \ell}{\partial \alpha} = \alpha^{-1} \sum_{i=1}^{n} \delta_i + \ln \lambda \sum_{i=1}^{n} \delta_i + \sum_{i=1}^{n} \delta_i \ln u_i - \lambda^{\alpha} \ln \lambda \sum_{i=1}^{n} u_i^{\alpha} - \lambda^{\alpha} \sum_{i=1}^{n} u_i^{\alpha} \ln u_i.$$

and that for λ is:

$$\mathcal{U}_\lambda = \frac{\partial \ell}{\partial \lambda} = \alpha \lambda^{-1} \sum_{i=1}^{n} \delta_i - \alpha \lambda^{\alpha - 1} \sum_{i=1}^{n} u_i^{\alpha},$$

The sample-level observed information matrix is:

$$\mathcal{J}_n = - \begin{pmatrix} \mathcal{H}_{\alpha\alpha} & \mathcal{H}_{\alpha\lambda} \\ \mathcal{H}_{\lambda\alpha} & \mathcal{H}_{\lambda\lambda} \end{pmatrix},$$

where

$$\mathcal{H}_{\alpha\alpha} = \frac{\partial^2 \ell}{\partial \alpha^2} = -\alpha^{-2} \sum_{i=1}^{n} \delta_i - \lambda^{\alpha} (\ln \lambda)^2 \sum_{i=1}^{n} u_i^{\alpha} - 2\lambda^{\alpha} \ln \lambda \sum_{i=1}^{n} u_i^{\alpha} \ln u_i - \lambda^{\alpha} \sum_{i=1}^{n} u_i^{\alpha} (\ln u_i)^2$$

$$\mathcal{H}_{\alpha\lambda} = \frac{\partial^2 \ell}{\partial \alpha \partial \lambda} = \lambda^{-1} \sum_{i=1}^{n} \delta_i - \lambda^{\alpha - 1} \sum_{i=1}^{n} u_i^{\alpha} - \alpha \lambda^{\alpha - 1} \ln \lambda \sum_{i=1}^{n} u_i^{\alpha} - \alpha \lambda^{\alpha - 1} \sum_{i=1}^{n} u_i^{\alpha} \ln u_i$$

$$\mathcal{H}_{\lambda\lambda} = \frac{\partial^2 \ell}{\partial \lambda^2} = -\alpha \lambda^{-2} \sum_{i=1}^{n} \delta_i - \alpha(\alpha - 1) \lambda^{\alpha - 2} \sum_{i=1}^{n} u_i^{\alpha}.$$

The standard error of the Weibull median depends on the expected information matrix of $(\alpha, \lambda)'$. Calculation of the expected information $\mathcal{I}_n = \mathbb{E}(\mathcal{J}_n)$ requires knowledge of the distribution of U_i, which in turn depends on the distribution of censoring times C_i. Even in the simple setting of exponentially distributed censoring times, the distribution of $U_i = \min(T_i, C_i)$ is challenging to work with. Therefore, to estimate $\mathcal{I}_n$, we proceed as follows. Let π_C denote the *expected censoring proportion*, defined as $\pi_C = \mathbb{P}(T > C)$. For $T_i \sim \text{Weibull}(\alpha, \lambda)$, simulate Weibull censoring times $C_i \sim \text{Weibull}(\alpha, \delta)$ with the same shape parameter α, and a rate parameter δ set to:

$$\delta = \left(\frac{\pi_C}{1 - \pi_C}\right)^{1/\alpha} \lambda.$$

It is easy to check that with this choice of censoring distribution, the expected censoring proportion is indeed π_C. To estimate $\mathcal{I}_n$, we generate a small number n_{info} of data sets (e.g. 50), at sample size n, with $T_i \sim \text{Weibull}(\alpha, \lambda)$, $C_i \sim \text{Weibull}(\alpha, \delta)$, $U_i = \min(T_i, C_i)$, and $\delta_i = \mathbb{I}(T_i \leq C_i)$. For the rth simulated data set, let $\mathcal{J}_{n,r}$ denote the sample-level observed information. A consistent estimate of the sample-level expected information is given by:

$$\hat{\mathcal{I}}_n = \frac{1}{n_{\text{info}}} \sum_{r=1}^{n_{\text{info}}} \mathcal{J}_{n,r}.$$

Given data $\{(U_i, \delta_i\}_{i=1}^{n}$, let $\hat{\alpha}$ denote the maximum likelihood estimate (MLE) of the shape parameter α, and $\hat{\lambda}$ the MLE of the rate parameter λ. From standard maximum likelihood theory:

$$\sqrt{n} \begin{pmatrix} \hat{\alpha} - \alpha \\ \hat{\lambda} - \lambda \end{pmatrix} \rightsquigarrow N\big(\mathbf{0}, i^{-1}\big),$$

where i is the limit in probability of $n^{-1}\mathcal{I}_n$. The median of a Weibull(α, λ) distribution is

$$\mu(\alpha, \lambda) = \lambda^{-1}(\ln 2)^{1/\alpha}$$

The gradient of the median with respect to $(\alpha, \lambda)'$ is:

$$\nabla\mu = \begin{pmatrix} \frac{\partial\mu}{\partial\alpha} \\ \frac{\partial\mu}{\partial\lambda} \end{pmatrix} = \begin{pmatrix} -\lambda^{-1}(\ln 2)^{1/\alpha}(\ln\ln 2)\alpha^{-2} \\ -\lambda^{-2}(\ln 2)^{1/\alpha} \end{pmatrix}$$

Letting $\hat{\mu} = \mu(\hat{\alpha}, \hat{\lambda})$ denote the MLE of the median, then by the Δ method:

$$\sqrt{n}(\hat{\mu} - \mu) \rightsquigarrow N\big(0, \nabla\mu' i^{-1} \nabla\mu\big),$$

The standard error of the median is readily estimated via:

$$\hat{\sigma}_\mu^2 = \mathbb{V}(\hat{\mu}) = \nabla\hat{\mu}'\hat{\mathcal{I}}_n^{-1}\nabla\hat{\mu},$$

where $\nabla\hat{\mu}$ denotes the gradient of μ evaluated at $(\hat{\alpha}, \hat{\lambda})$, and $\hat{\mathcal{I}}_n$ is the sample-level expected information, estimated as described above.

2 Additional Simulation Studies

The following simulation studies compare the analytical selection probability calculation, based on the asymptotic results discussed above and in the main text, with an empirical selection probability calculation. The empirical calculation was performed by averaging an indicator $\mathbb{I}(\hat{\mu}_2 - \hat{\mu}_1 > \text{MPE})$ plus $0.5 \times \mathbb{I}(|\hat{\mu}_2 - \hat{\mu}_1| \leq \text{MPE})$ across the $R = 10^3$ simulated data sets.

2.1 No Difference

Figure 1 examines the selection probability calculation in the case of two equivalent exponential distributions, and Figure 2 in the case of two equivalent Weibull distributions (shape parameter: $\alpha = 2$). In each case the rate parameter was selected such that the median time to event was 6 months. Censoring rates of 0% and 20% are considered, and selection margins of 0 and 2 months. The analytical calculation in red agrees well with the theoretical value of 0.5.

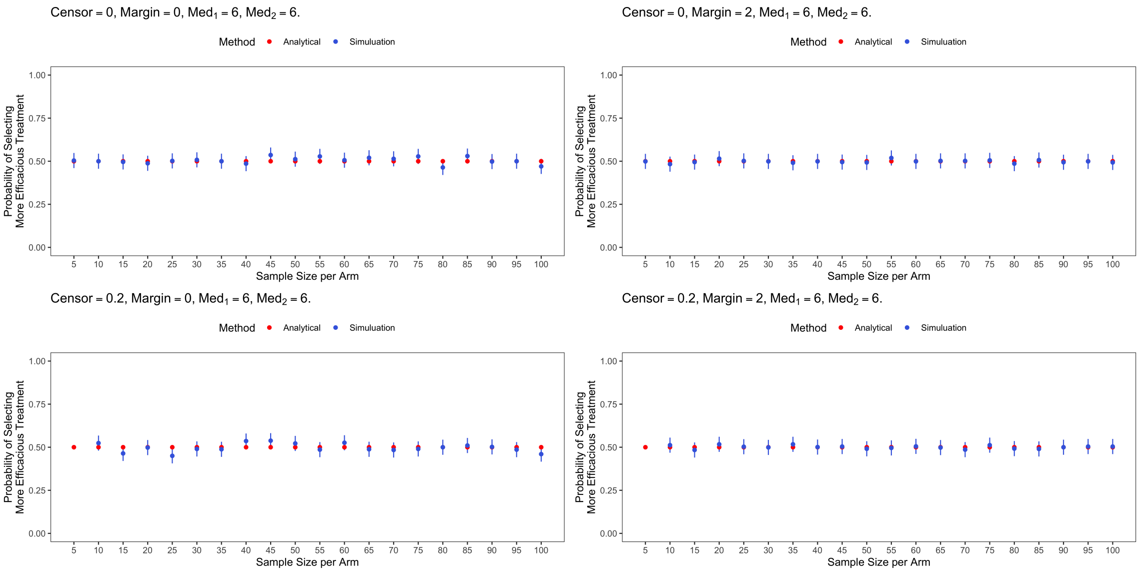

Figure 1: Selection Probability by Sample Size for Two Equivalent Exponentials.

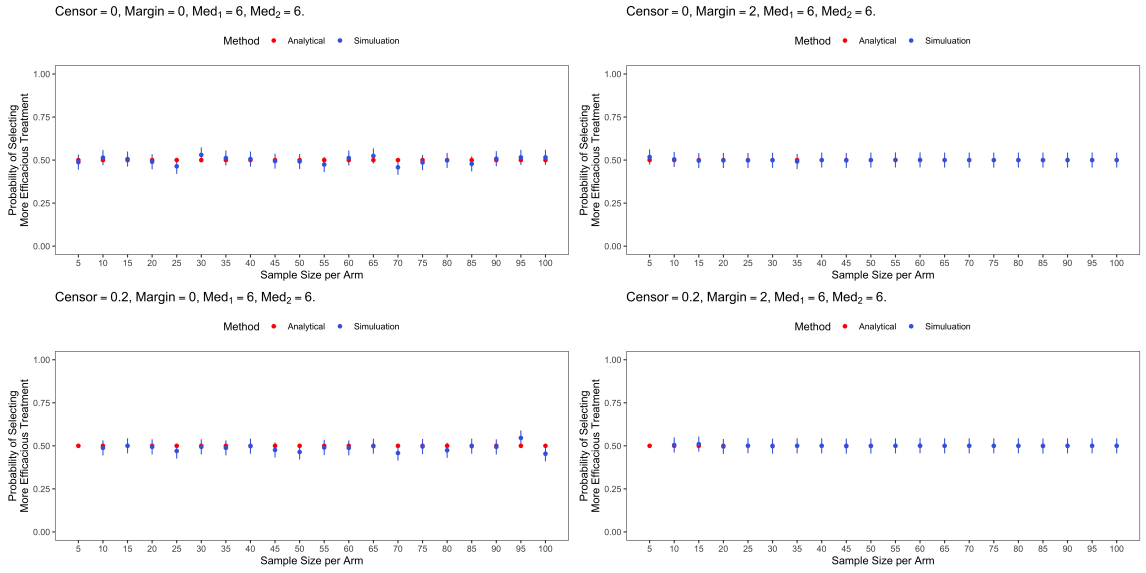

Figure 2: Selection Probability by Sample Size for Two Equivalent Weibulls.

2.2 1 Month Difference

Figure 3 examines the selection probability calculation for two exponential distributions with median event times of 6 and 7 months, respectively. Figure 4 considers the analogous case for two Weibull distributions (shape parameter: $\alpha = 2$). Censoring rates of 0% and 20% are examined, and selection margins of 0 and 2 months. The analytical calculation in red agrees well with the empirical calculation in blue.

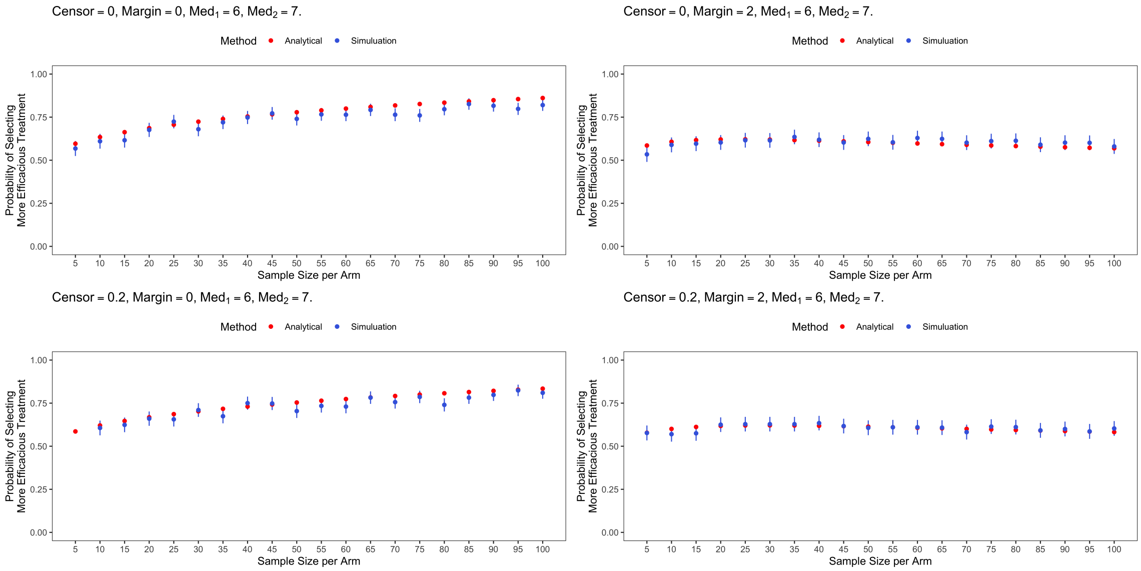

Figure 3: Selection Probability by Sample Size for Two Exponentials with Median Event Times Differing by 1 Month.

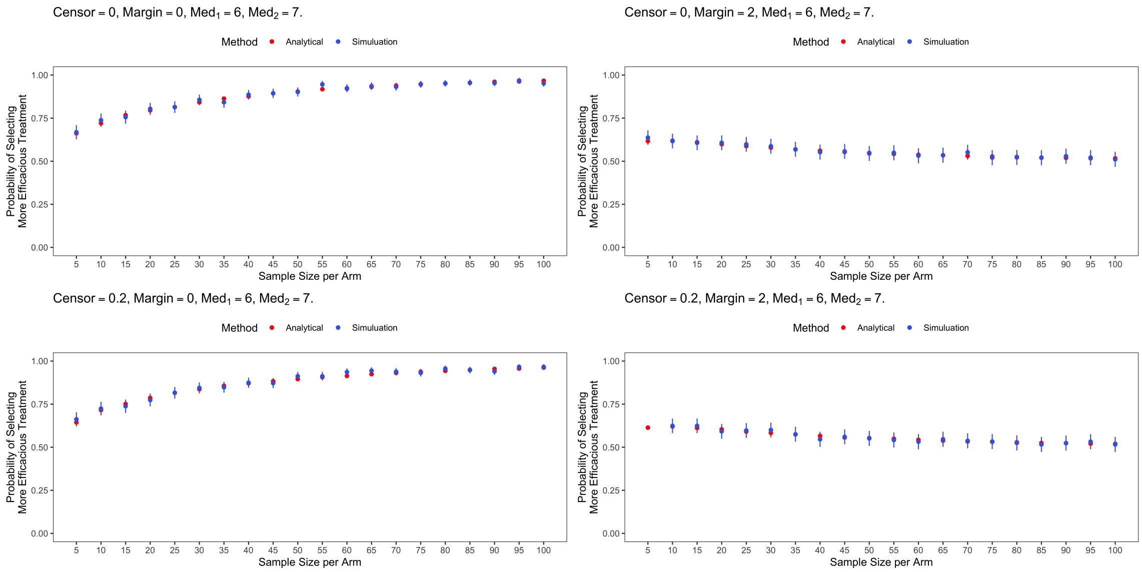

Figure 4: Selection Probability Calculation by Sample Size for Two Weibulls with Median Event Times Differing by 1 Month.

2.3 3 Month Difference

Figure 5 examines the selection probability calculation for two exponential distributions with median event times of 6 and 9 months, respectively. Figure 6 considers the analogous case for two Weibull distributions (shape parameter: $\alpha = 2$). Censoring rates of 0% and 20% are examined, and selection margins of 0 and 2 months. The analytical calculation in red agrees well with the empirical calculation in blue.

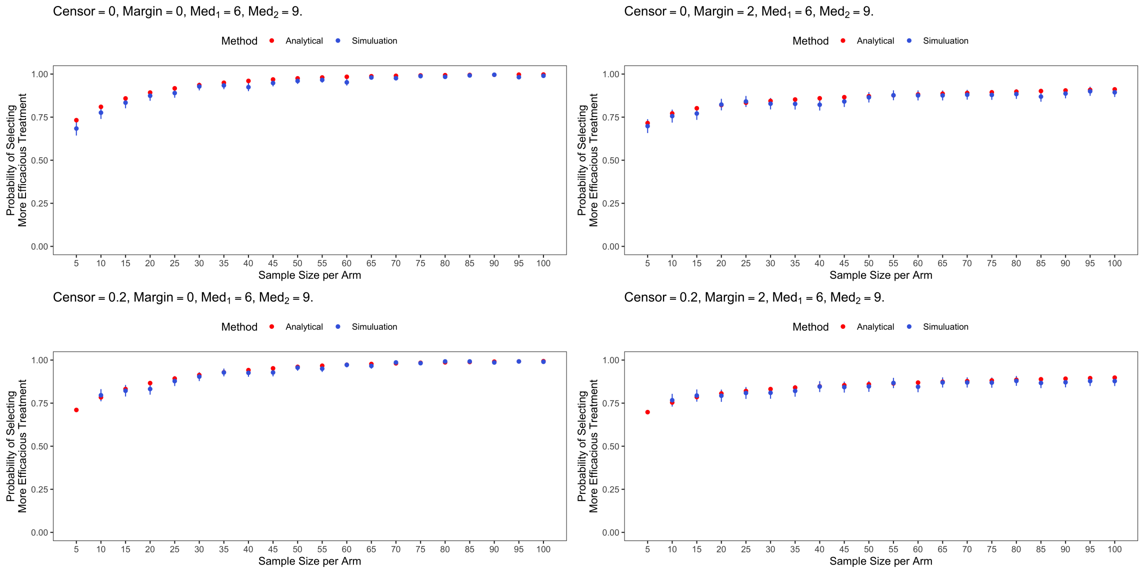

Figure 5: Selection Probability by Sample Size for Two Exponentials with Median Event Times Differing by 3 Months.

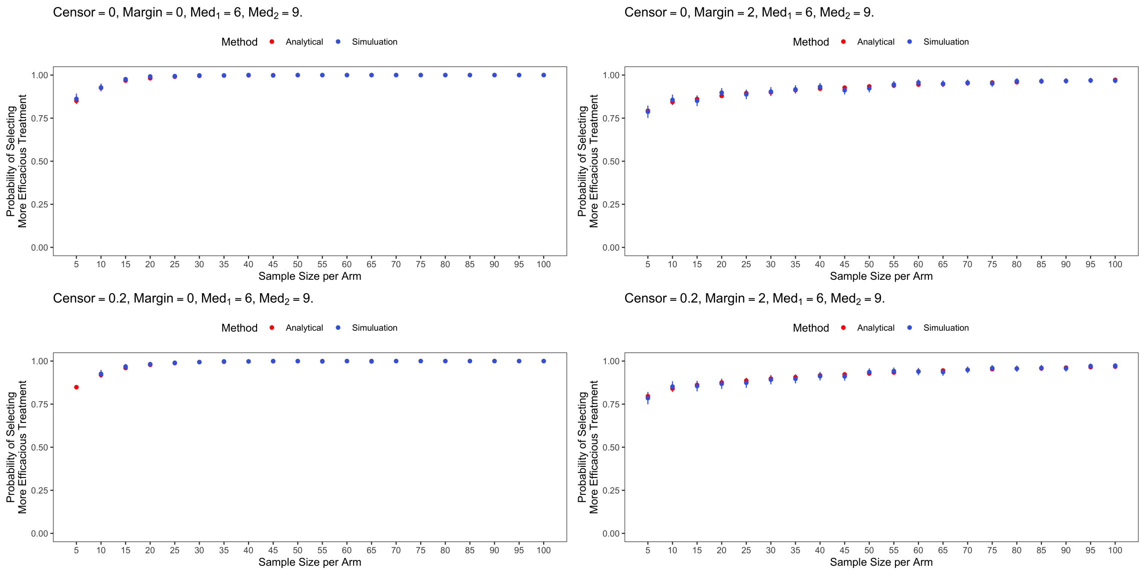

Figure 6: Selection Probability Calculation by Sample Size for Two Weibulls with Median Event Times Differing by 3 Months.
